# Non-nominal Deployment of the SAPIEN 3 Transcatheter Heart Valve: An Ex Vivo Bench Study

**DOI:** 10.1016/j.jscai.2023.101217

**Published:** 2023-11-27

**Authors:** Hari P. Sritharan, Kunwardeep S. Bhatia, Bipeen Gautam, Nadeem Mughal, Avedis A. Ekmejian, Usaid K. Allahwala, Ravinay Bhindi, Peter S. Hansen

**Affiliations:** a Royal North Shore Hospital, St Leonards, New South Wales, Australia; b University of Sydney, Sydney, New South Wales, Australia; c Bankstown-Lidcombe Hospital, Sydney, New South Wales, Australia

**Keywords:** aortic valve stenosis, heart valve prosthesis, transcatheter aortic valve replacement

## Introduction

The SAPIEN 3 (S3) (Edwards Lifesciences) is the most used balloon-expandable valve in transcatheter aortic valve replacement. The manufacturer recommends filling the deployment balloon to nominal volume only.^1^ However, this can lead to a mismatch between the patient’s native aortic annulus area and the nominal S3 cross-sectional area.^2^ Undersizing the valve can lead to paravalvular leak, valve migration/embolization, and patient prosthesis mismatch while oversizing can lead to increased permanent pacemaker rates and aortic annulus rupture.^1^^,^^3^ Non-nominal deployment of the S3 is common in clinical practice, although not recommended by the manufacturer. Current off-label sizing charts assume a 1:1 relationship between the deployment balloon filling volume and the S3 cross-sectional area. However, there exists no bench-tested sizing chart to accurately guide clinicians on non-nominal deployment. We aimed to assess the expansion of the 23, 26, and 29 mm S3 at non-nominal deployment volumes in an ex vivo bench study.

## Methods

**Figure 1:**
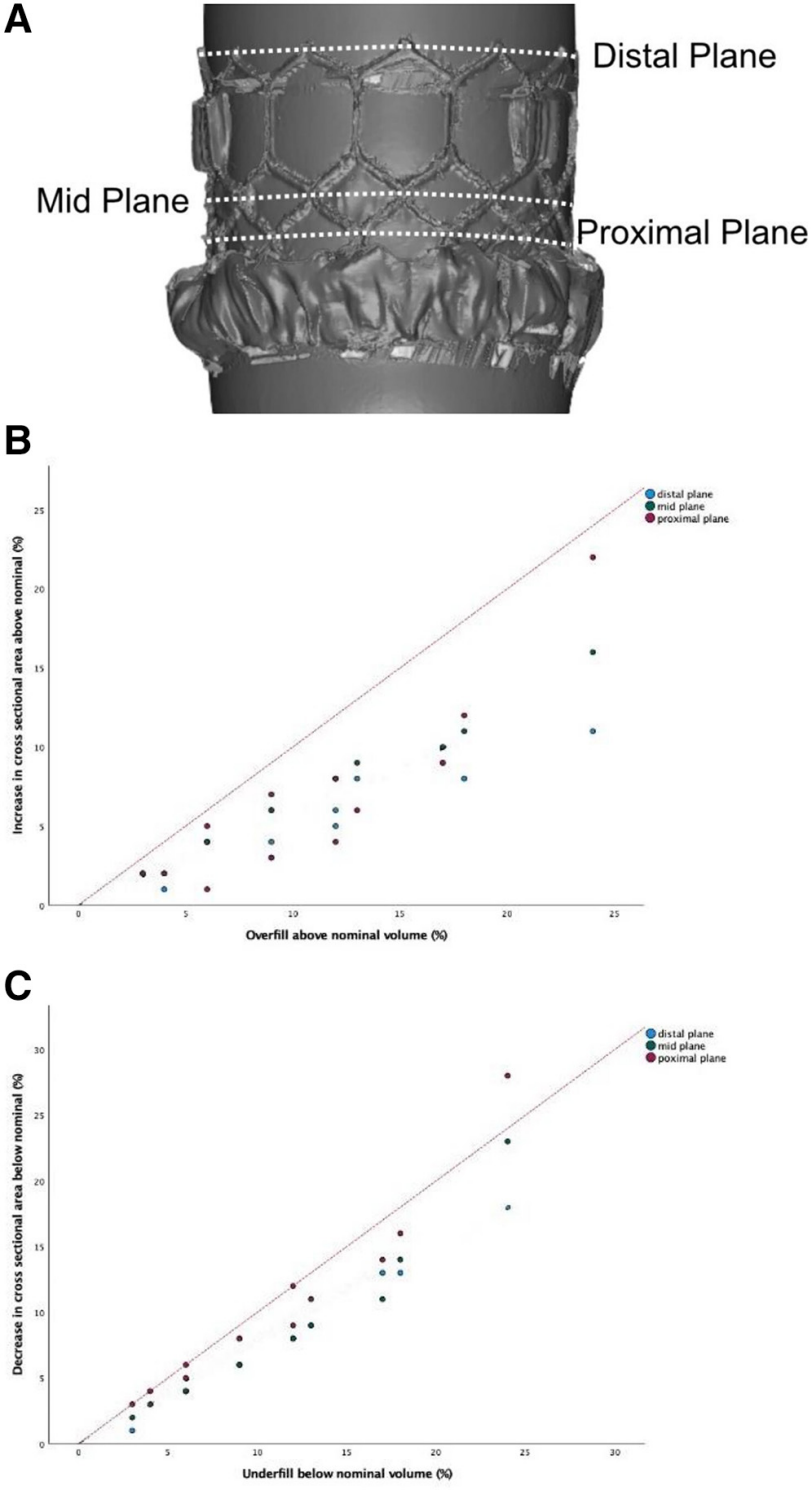
**Measurement planes and change in cross-sectional area with overfilling and underfilling.** (**A**) Measurement planes. The distal plane was defined as the top of the S3. The mid plane was defined as the midline of the upper row of diamond cells in the S3 frame. The proximal plane was defined as the midline of the lower row of diamond cells in the S3 frame. (**B**) Change in cross-sectional area with overfilling. Percentage overfill plotted against percentage increase in cross-sectional area. The dotted red lines represent the predicted 1:1 linear relationship. (**C**) Change in cross-sectional area with underfilling. Percentage underfill plotted against percentage decrease in cross-sectional area. The dotted red lines represent the predicted 1:1 linear relationship.

The manufacturer-provided S3 inflation device was filled with diluted radiopaque contrast (15:85 ratio of contrast to saline) and locked. The S3 was mounted and crimped onto the Commander Delivery System (Edwards Lifesciences) using standard procedures. The delivery system was suspended on 2 stands of equal height using the stylet in the guide wire lumen of the delivery system, then slow controlled inflation of the balloon was performed to 4 mL less than the nominal volume. The balloon and valve were then coated with ammonium chloride vapor to enable 3-dimensional scanning. Three-dimensional scanning was performed using the 2.5 meter Kreon Ace+ 7 Axis 3D scanning arm with a Skyline Eyes Scanner (Europac 3D). After each scan, incremental 1 mL balloon inflations were performed until overfilling by 4 mL with measurement at 3 different cross-sectional planes (Figure 1A). Three-dimensional renderings were analyzed using Geomagic Wrap and Geomagic Control (3D Systems Inc) and Rhinoceros (Robert McNeel and Associates). Statistical analysis was performed using SPSS Statistics Subscription build 1.0.0.1461 (IBM Corp). Linear fit lines (least squares method) with intercept suppression were utilized to explore trends in the data.

## Results

The expansion of the S3 was non-uniform. At all deployment volumes, the valve was waisted at the midplane. The minimum variation between the distal plane and midplane was 5% (26 mm S3 at 9% overfilling) and the maximum variation was 16% (23 mm S3 at 24% underfilling). At nominal inflation, the maximum diameter at the proximal plane (zone which interacts with the aortic annulus) aligned closely with the labeled diameter for each valve (22.7 mm for 23 mm S3, 25.9 mm for 26 mm S3, and 29.0 mm for 29 mm S3).

When the deployment balloon was overfilled, the percentage increase in S3 cross-sectional area was less than the percentage increase in filling volume at all planes. The proximal plane (most responsive to overfilling) increased by 4% when overfilled by 12% in the 23 mm S3, 6% when overfilled by 13% in the 26 mm S3, and 8% when overfilled by 12% in the 29 mm S3. The distal plane was the least responsive to changes in filling volume when overfilled (Figure 1B).

When the deployment balloon was underfilled, the distal and midplane cross-sectional area of the S3 decreased variably compared to the filling volume. However, the proximal plane was highly responsive to underfilling. When underfilled by <10% the proximal plane reduced in cross-sectional area within 1% of the percentage change in filling volume. Overall, the percentage decrease in cross-sectional area for each valve was less than the percentage decrease in filling volume (Figure 1C).

## Discussion

Our results demonstrate that the filling volume of the deployment balloon does not have a 1:1 linear relationship with the cross-sectional area of the S3. These results are contrary to current off-label sizing charts that assume an approximate 1:1 relationship.^4^ This deviation is more pronounced when overfilling, rather than underfilling the deployment balloon. When underfilling the deployment balloon, the S3 performs in a manner closer to the assumed 1:1 linear relationship. The expansion of the S3 is also non-uniform, with different parts of the stent frame responding differently to underfilling and overfilling. This has important implications for operators trying to “right size” the S3, as the implantation depth and coaxiality will determine the part of the S3 stent frame that is in contact with the aortic annulus and thus the degree of change in cross-sectional area at the level of the annulus that will be achieved with a certain amount of overfilling. Interventionalists should be aware that non-nominal deployment of the S3 is off-label and is specifically discouraged in the manufacturer’s instructions for use. Further investigation is needed to assess leaflet coaptation and hemodynamics at clinically relevant degrees of overexpansion.

Our study has the inherent limitations of ex vivo bench testing, as the valve may behave differently in vivo. The impedance of expansion by aortic calcification was not modeled in our bench study; however, even the manufacturer-provided nominal sizing charts do not account for this. We also do not account for the slight recoil in the S3 stent frame with balloon deflation,^5^ changes in pressure with a change in filling volume, and our mode of incremental expansion differs from in vivo practice. Furthermore, only 1 run of each valve was performed, and slight variability between valves remains possible. The proximal plane in our study is approximately one-third of the height of the valve. While this is an acceptable implantation depth, interventionalists commonly aim for 80:20 or 90:10 aortic:ventricular implantation depth. The cross-sectional area was not measured at these heights, as the fabric skirt obscured measurement. In the context of these limitations, the exact values assessed should not be used as a sizing chart, rather they should inform the interventionalist that the relationship between overfilling and cross-sectional area is not 1:1 and highlight the need for further research in this area.

## Conclusion

Percentage changes in filling volume translate to smaller percentage changes in the cross-sectional area when deploying the S3 at clinically relevant non-nominal volumes.
